# Analysis on the potential of *Pennisetum hydridum* for phytoremediation of Cd-polluted soil fertilized by worm castings

**DOI:** 10.1371/journal.pone.0318528

**Published:** 2025-03-31

**Authors:** Xiafang Hong, Zhi Zhang, Zhiwei Wan

**Affiliations:** 1 Yuzhang Normal University, Nanchang, China,; 2 Shandong Provincial Key Laboratory of Water and Soil Conservation and Environmental Protection, College of Resources and Environment, Linyi University, Linyi, China; Kobe University: Kobe Daigaku, JAPAN

## Abstract

Soil heavy metal pollution including Cd, is the main factor that causes the decline of ecological environment quality, the excessive content in crops and the harm to human health. Phytoremediation is one of the important ways to control heavy metals, which has both ecological and economic benefits. However, most plant species have limited remediation ability and cannot achieve good heavy metal removal effect. In contrast, *P. hydridum*, easy to cultivate, has large biomass and short growth cycle, shows strong restoration ability in the treatment of heavy metal polluted soil. In order to explore its phytoremediation in Cd-polluted soils under appropriate agronomic measures, this experiment adopts the field random block experiment design to study the control effect and application safety of the application of organic fertilizers (warm castings and biogas slurry) to plant it in the Cd-polluted farmland. The results showed the Cd in the soil after *P. hydridum* harvesting was 0.53–0.56 mg/kg, and the partial Cd in the shoot was 0.21–0.28 mg/kg (fresh weight), and the enrichment coefficients were all greater than 1, and the extraction amount and efficiency of Cd were 7.17–9.43 mg/m^2^ and 5.71%–7.01%, respectively. All those data express a decrease in Cd under various treatment conditions, indicating that, *P. hydridum* can grow under high concentration and it has a certain enrichment effect on Cd, especially in the application of organic fertilizers, which could not only improve the growth performance of the plant, but also improve the soil, much better than that of other Cd hyper-accumulators. Moreover, the positive correlation between the biomass allocation rate and Cd in the soil reflects that the biomass allocation of the plant behaved in different ways with the increase of Cd oil. It is also feasible in terms of application safety due to a long and gradual process to enrich Cd in soil. This study made a proof that it would be a green and environmentally friendly treatment method by making good use of its high biomass to adsorb and remove heavy metals from soil, which would have a good application prospect and development value.

## Introduction

Soil heavy metal pollution is the main factor that causes the decline of ecological environment quality, the excessive content of heavy metals in crops and the harm to human health. Cd is a non-essential element [1,2] that has adverse effects on plant growth [3–9]. Even a small amount of daily Cd intake can cause a large amount of Cd accumulation in the human body under long-term conditions, causing irreversible damage [10–14]. The International Agency for Research on Cancer IARC also lists Cd as a Class 1 carcinogen in humans. It is one of the most dangerous heavy metal elements in agricultural environment, as it can inhibit plant growth, change plant morphology, and affect plant physiological and biochemical and structure [15]. Moreover, it has a cumulative effect, damaging human health through the food chain [16]. On April 17, 2014, China’s Ministry of Environmental Protection and Ministry of Land and Resources issued the National Soil Pollution Survey Bulletin (http://www.ndrc.gov.cn/fzgggz/ncii/zhdt/201404/t20140418607888.html), which shows the results of a survey on 6.3 million km^2^ of soil in China from 2005 to 2013, covering all cultivated land, as well as some potentially cultivatable forest land, grassland, unused land, and construction land. In China, by the end of 2013, the total over-standard rate of heavy metal pollution was 16.1%, and inorganic pollutants account for 82.8% of the total over-standard points. The pollution levels of Cd, mercury (Hg), arsenic (As), and lead (Pb) gradually increased from north to south, and the over-standard rates were 7.0%, 1.6%, 2.7%, and 1.5%, respectively. Furthermore, one sixth of the total cultivated land area in China had an over-standard rate of cultivated soil sites of 19.4%. Among the inorganic pollutants, Cd exceeded the standard rate of 7.0% in these sites, making it the most abundant element within them. In typical plots such as heavily polluted commercial land, industrial waste land, and mining areas and their surroundings, the excess rate of Cd can exceed 30%.On June 22, 2018, the Ministry of Ecology and Environment of China and the State Administration of Market Supervision and Administration released the GB156182018 Soil Environmental Quality Agricultural Land Soil Pollution Risk Control Standard (for Trial Implementation) (Ministry of Ecology and Environment and State Administration of Market Supervision and Administration, 2018). Under China’s actual situation and the environmental chemical behavior characteristics of Cd in soil, screening values and risk control levels were proposed for different types of agricultural land. Taking paddy field as an example, when pH ≦ 5.5, the risk screening value of Cd content is less than 0.3 and the risk control value is less than 1.5 mg/kg. When 5.5 < pH ≦ 6.5, the screening value is less than 0.4 and the control value is less than 2.0 mg/kg. When 6.5 <  pH ≤  7.5, the screening value is less than 0.6 mg/kg and the control value is less than 3.0 mg/kg. When pH > 7.5, the screening value is less than 0.8 mg/kg and the control value is less than 4.0 mg/kg. It can be seen when the Cd content in soil is lower than the risk screening value, the risk of soil pollution in agricultural land can be considered low and can usually be ignored. When the Cd content in soil exceeds the risk screening value but lower than or equal to the risk control value, there may be risks such as edible agricultural products not meeting quality and safety standards. In principle, corresponding agronomic control and alternative planting measures should be taken to ensure the safe utilization of soil. When the soil Cd content is higher than the risk control value, there may be risks such as edible agricultural products not meeting quality and safety standards. In principle, corresponding agronomic control and alternative planting measures should be taken to ensure the safe utilization of soil.

Phytoremediation is one of the important ways to control heavy metals, which has both ecological and economic benefits. However, most plant species have limited remediation ability and cannot achieve good heavy metal removal effect. Whether phytoremediation technology can be widely used in heavy metal pollution control projects and provide scientific support for remediation of contaminated soil and restoration of the soil ecological environment depends on plant growth, scope of application, and remediation efficiency [17]. The preferred plants to resist heavy metal pollution are mainly grasses and shrubs [18]. At present, the plants chosen in soils have the problems, such as low biomass, narrow application range, little sourcing, and long restoration period. For example, the southeast sedum cannot grow in the cold northern region and is difficult to oversummer in the southern region. *Solanum*, *Artemisia* S*elengensis*, *Bidens pilosa*, and other plants are difficult to source after harvest. The long growth cycle and slow growth speed of tree species and plants are not suitable for urgent restoration work, which limit the application of phytoremediation in the treatment of Cd polluted cultivated land [19]. The long growth cycle and slow growth speed of tree species and plants are not suitable for urgent restoration work, which limit the application of phytoremediation in the treatment of Cd polluted cultivated land [20]. In addition, the remediation efficiency of plants is not only related to the biomass, growth cycle, and tolerance to heavy metals, but is also affected by a series of factors such as climate, moisture, soil pH, organic matter content, and soil microorganisms. Therefore, it is urgent to identify a rehabilitative plant with strong adaptability, short growth cycle, and availability for sourcing to reduce the damage of Cd pollution in Chinese soil. In contrast, forage grass is easy to cultivate, with large biomass and short growth cycle. It shows strong remediation ability in the treatment of heavy metal contaminated soil, and has good application prospect and development value. Because of the limited cultivated land area in China, it takes time to use phytoremediation to remediate soil. Therefore, not only can the extraction efficiency of plants be improved through the application of common crops or changing the conditions of rhizosphere sharing, but farmers can continue to engage in agricultural activities, forming an efficient planting and restoration system involving concurrent remediation and production. At present, phytoremediation has many limitations. Therefore, economic remediation plants are selected based on local conditions and the degree of heavy metal pollution, corresponding efficiency measures are provided to shorten the remediation period of paddy fields, and the strategic goal of prevention and control and reduction of Cd pollution in farmland soil is realized, which is of great importance to food safety and ecological security.

*P. hydridum*, a perennial upright bunching grass of the genus *Pennisetum*, is hybridized and bred from diploid *American pennisetum* as the mother and tetraploid *Pennisetum Purpureum Schum*, so also known as *hybrid Pennisetum*, a triploid C4 plant. It is a kind of a high-yield and high-quality cutting type forage, simple planting, fast growth rate, wide adaptability, strong stress resistance, high biomass, rich nutrition, and also. In recent years, it is widely used in feed, paper, beverage and food and other fields. The large biomass of this herb means that it has considerable potential for remediation of heavy metal contaminated soil [21]. Studies have shown that the application of functional soil remediation agents significantly improves the physical and chemical properties of soil [22]. The use of worm castings to improve soil can not only realize the resource utilization of manure, but also serve as a key source for improving soil fertility and accelerating the process of soil improvement by containing a large amount of organic matter, humic acid and lumbrokinase [23].

Based on the experimental tests on the literature and considerations, in this study, *P. hydridum* was selected to plant under the different field treatments with the application of organic fertilizers (castings and biogas slurry), to study the effect and the change in Cd content in soil and effect of pH value on biomass, the effects of Cd on biological indicators and the safety of its subsequent application was analyzed [24]. It will provide reference for the treatment, restoration and safe application of Cd- pollution soil. In addition, its main economic and technical indicators are high, if the safety performance is studied when it is used for pollution control, the potential in solving heavy metal pollution and supporting sustainable agricultural practices, to help better understand the characteristics and advantages of the ecological management and application prospects.

## Materials and Methods

### Overview of the test area

The experimental area is located at the *P. hydridum* Base of *Jinnong Animal Husbandry Eco-agriculture Family Farm*, *Zhutian Township*, *Suichuan* county, *Ji’an* city, *Jiangxi* Province (25°28’32”–26°42’55”N, 113°56’51”–114°45’45”E). It is a humid monsoon climate in the middle subtropical zone, with mild climate, abundant rainfall, abundant sunshine, four distinct seasons (long winter and summer, short spring and autumn), a long frost-free period, and variable climate. The annual average temperature is between 15.1 and 18.1°C, the annual average precipitation is 1,400 mm, and the annual average frost-free period is 284 days. The paddy land is fertile, and the mountains are red soil.

The soil conditions in the test area are as follows: the pH is 6.26 ±  0.20, which is acidic, and the total soil Cd is 0.61 ±  0.20 mg/kg, which exceeds the second class limit of GB 15618-1995 (total soil Cd is less than 0.3 mg/kg; pH is less than 6.5; organic matter is 2.41 ± 0.20; and N, P, and K are 2.38 ± 0.20, 74.21 ± 0.20, and 115.42 ± 0.20, respectively), indicating slightly Cd polluted status.

### Materials

*P. hydridum* seedlings were provided by the local golden agriculture and animal husbandry ecological agriculture demonstration park. On May 18, 2022, seedlings were selected and raised for approximately 30 days in the same planting field, with relatively consistent growth, plant height of 35–40 cm, true leaf number of 4–5, no tillers, and no difference in specifications. Two complete stem segments were retained as test material. Lime originated from local the lime mill, with a *pH* of 13 and total Cd of 0.21 mg/kg. Sodium hydroxide originated from *Xinjiang Zhongtai Chemical Co., Ltd.*, and the total Cd content was 1.19 mg/kg. Warm castings were provided by *Ganzhou JASPER Earth-worm Castings Co., Ltd.,* pH 7.69, total Cd content 1.09 mg/kg. Biogas slurry: produced from sewage of local cattle farm.

### Test design

#### Field planting.

Two plots were set up as a field positioning experiment: one was planted and the other was not planted. The area of each plot was approximately 667 m^2^ and the planting area was 10 hm^2^, which was convenient for the workload of a harvester. Two groups of soil additive application treatment were arranged in each area: (1) no soil additive application (control), (2) additive application (worm castings as a base fertilizer, 20 kg/ m^2^ and continuous application of biogas slurry). Each group of treatments was repeated twice, and the orientation of each treatment was randomly distributed in the cell. The soil in the experimental area was leveled, ploughed, and divided in November of 2021, and the additive was applied. On May 18, 2022, the seedlings were transplanted for approximately 30 days. In order to facilitate mechanized harvesting, the planting density (row spacing ×  plant spacing) was 80 cm ×  60 cm, 130 seedlings per mu, and an average of 4 seedlings/m^2^. The test period was 6 months (May to November of 2022). No mowing was carried out during the test period, and no fertilization was carried out during the whole growth period of plants. The irrigation water met the “Quality Standard for Farmland Irrigation” (GB 5084-2005) (Ministry of Agriculture of the PRC, 2005). The field weeds were removed by artificial weeding. Considering that the research area is located in a rural area of Jiangxi Province with good air quality, heavy metal pollution caused by air deposition is temporarily not considered.

#### Method for sample determination.

Six months later (Nov. of 2022), since no cutting was carried out during this period, the basic height of the plants was more than 2 m, the diameter of each plant was approximately 60 cm on average, and the number of tillers exceeded 30. In each area, the five-point method was used to sample four plants within 1 m^2^ of each sample point, the aboveground and underground parts of the plant body were opened, and the whole bag was dug out, sampled, and measured. The remaining plants were crushed and harvested by a harvester. The shoot comprised green leaves, withered yellow leaves (2/3 or more withered yellow), green leaf sheath, withered leaf sheath, and stem, which constituted the mixed sample. The soil adhered to the surface was removed with tap water at the lower part of the ground, rinsed with deionized water 3 times, and air dried. Part of the sample was selected for drying (After 30 min de-enzymization at 105°C, it was dried to constant weight at 65°C) and used to determine the dry matter quantity of each part. After ploughing, soil and plants were harvested: soil samples were collected in each area according to the five-point method, and mixed samples of the 0–20-cm ploughing layer soil were taken and numbered at the sampling sites. The samples were air-dried indoors, and stones, plant roots, and soil litter were removed and crushed. A 30-mesh (0.6 mm) sieve was used to determine the soil Cd content. The soil samples were boiled in HNO_3_-H_2_O_2_, and the plant samples were boiled in HNO_3_-HClO_4_ (Ministry of Health of the PRC, 2003). The GB/T 5009.15-2003 method was used for the determination of heavy metal Cd in soil, and Cd was computed by atomic adsorption spectroscopy.

#### Evaluation of remediation effectiveness.

To evaluate the remediation effect, plant extraction amount, plant extraction efficiency, and estimated restoration period were used for comparison and analysis. The calculation formulas were as follows:


$${\text{P}}_{\text{i}}{\text{=C}}_{\text{i}}{\text{·W}}_{\text{i}}\text{,}$$ (1)

$${\text{Q}}_{\text{i}}{\text{= ρ}}_{\text{b}}{\text{·S}}_{\text{i}}{\text{·H}}_{\text{i}}\text{,}$$ (2)

$${\text{EE}}_{\text{plant}}{\text{= P}}_{\text{i}}\text{/ }\left({\text{C}}_{\text{i}}{\text{·Q}}_{\text{i}}\right)\text{·100,}$$ (3)

$${\text{BAF = C}}_{\text{i}}{\text{/C}}_{\text{1}}\text{,}$$ (4)

$${\text{A}}_{\text{i}}{\text{= Q}}_{\text{i}}\text{· }\left({\text{C}}_{\text{1}}{\text{- S}}_{\text{1}}\right){\text{/P}}_{\text{i}}\text{,}$$ (5)

where *P*_*i*_ is the extraction amount of plant heavy metal Cd, mg/m^2^; *C*_*i*_ is the average concentration of plant heavy metal Cd, mg/kg dry weight; *W*_*i*_ is the dry weight of plants per crop, t/hm^2^; *Q*_*i*_ is soil mass, kg; ρ_b_ is the bulk density of soil, g/cm^3^; *S*_*i*_ is the planting area, m^2^; *H*_*i*_ is the soil thickness of the tillage layer, calculated as 20 cm; *EE*_*plant*_ is the extraction efficiency of heavy metal Cd, %; *C*_*1*_ is the average concentration of heavy metal Cd in soil before remediation, mg/kg; *BAF* is the enrichment coefficient, which means the grass has good enrichment if it is more than 1; *Ai* indicates the estimated repair time, a; and S1 is the national secondary standard limit value of soil environmental quality, mg/kg.

### Data processing

Data were analyzed by correlation and analysis of variance using Excel 2010 and SPSS 13.0 software according to Duncan’s test for significance (P <  0.05). The correlation model was fitted with the linear function, power function, exponential function, and binomial function. The model with the highest coefficient of determination *R*^*2*^ and significant correlation hypothesis test (α =  0.05) was selected for analysis and discussion. IBM SPSS 25 was used for data analysis, and two-factor ANOVA was used to compare the biomass, Cd enrichment characteristics, pH value, and organic acid content of the different treatments. Tukey’s test was used for multiple comparison, at a significance level of 0.05. If necessary, square root or log10 transformations were performed on the data to reduce the heterogeneity of the variance. Origin 2021 was used for mapping.

## Results

### Effect of Cd on *P. hydridum* growth under the different treatments

Under the first treatment condition, the biomass (fresh weight) of the grassland was 85.79–117.56 t/hm^2^ and no obvious stress occurred in the growth and development of the grassland. Compared with the control, the biomass was significantly increased by adding lime+sodium hydroxide and warm castings (P <  0.05), with an increase of 31.67 t/hm^2^.

**Fig 1:**
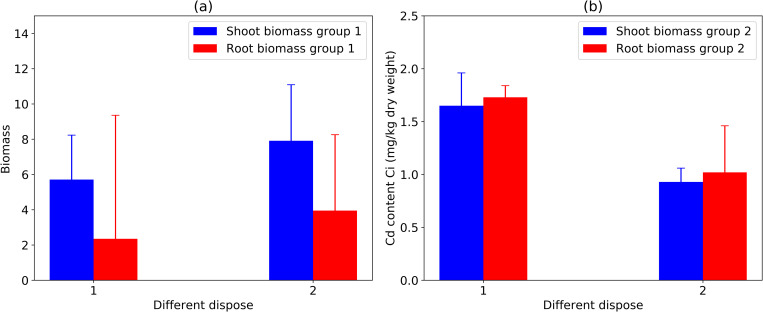
Biomass in *P. hydridum* with (2 of disposal) and without (1 of disposal).

As shown in Fig 1, under each treatment condition, the biomass (dry weight) of the shoot part was more than that of the root. Under treatment condition 1, the shoot was 5.71 ± 2.52 kg/m^2^, and the root was 2.35 ± 7.01 kg/m^2^. Under Treatment (2), the shoot is 7.91 ± 3.18 kg/m^2^, and the root is 3.95 ± 4.31 kg/m^2^. However, the average content of Cd in the boot is higher than that in the shoot. Under treatment condition 1, the shoot is 1.65 ± 0.31 mg/kg, and the root is 1.73 ± 0.11 mg/kg. Under Treatment (2), the Cd content concentration (*Ci*) of the shoot is 0.93 ± 0.13 mg/kg. The *Ci* of the root is 1.02 ± 0.44 mg/kg. Compared with the two treatment conditions, under Treatment (2), the content of Cd in *P. hydridum* was significantly decreased (P < 0.05). At the same time, although the biomass of the shoot is larger than that of the root, the Cd content, Cd extraction amount and *BAF* of the root are higher, indicating that the root has a stronger enrichment capacity for Cd. This was mainly due to the increase in the biomass of the *P. hydridum*, which also played a certain dilution role in the content of Cd in the plant. At the same time, the application of worm castings and biogas slurry [25] can increase soil fertility, change microbial community structure[26], and fix these in the soil through microbial meTableolism and the developed root system of the imperial *P. hydridum*, which reduces the availability of Cd in the soil and inhibits the absorption of Cd by the imperial. The availability of Cd in the soil was reduced after the application of additives, which inhibited the absorption of Cd by the grass.

**Fig 2:**
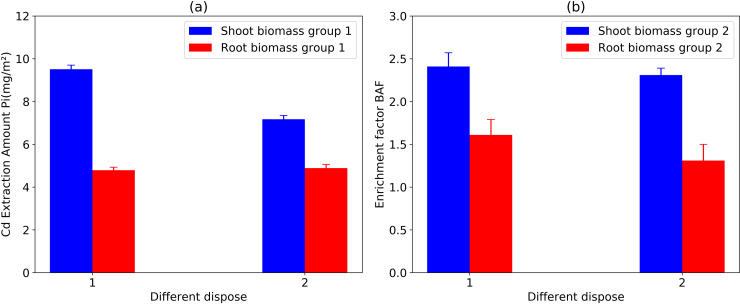
Cd extraction amount (*P*_*i*_) and enrichment factor (*BAF*) in *P. hydridum* with (2 of disposal) and without (1 of disposal).

As shown in Fig 2, under each treatment condition, the extraction *Pi* of Cd from *P. hydridum* was 9.51 ± 0.19 mg/m^2^ in the shoot and 4.79 ± 0.14 mg/m^2^ in the root. Under Treatment (2), the shoot was 7.17 ± 0.18 mg/m^2^, and the root was 4.89 ± 0.17 mg/m^2^. Under each treatment condition, the *BAF* value was greater than 1, showing certain Cd enrichment characteristics. Under Treatment (1), the *BAF* value was 2.41 ± 0.16 in the shoot and 1.61 ± 0.18 in the root. Under Treatment (2), the shoot is 2.31 ± 0.08, and the root was 1.31 ± 0.19.

### Change in Cd content in soil and effect of pH value on biomass

After the application of additives, the *P. hydridum* biomass increased, mainly because the alkaline additives slowed down soil acidity, regulated soil softness, and increased water and fertilizer use efficiency. In addition, soil organic matter provided sufficient nutrients and promoted plant growth. The shoot biomass was not significantly affected by soil Cd content and pH, whereas the root biomass was significantly affected by soil Cd content, and there was a significant interaction between soil Cd content and pH. The total biomass was significantly affected by soil Cd content, and there was a significant interaction between soil Cd content and pH (Table 1).

**Table 1:** Variance analysis of the effects of soil Cd content and pH on *P. hydridum* biomass.

Disposal	Total biomass	Shoot biomass	Root biomass
Cd	9.678**	0.135 ns	13.201***
pH	1.59 ns	0.251 ns	1.578 ns
pH × Cd	6.211**	2.47 ns	3.71 *

Note: The values in the Table are F values; * , P <  0.05; **, P <  0.01; ***, P <  0.001; ns, P >  0.05.

The average Cd content in the tillage soil before planting in the test area was 0.61 ± 0.21 mg/kg, which exceeds the limit value of Cd in the national secondary soil environmental quality standard (total soil Cd ≤ 0.3 mg/kg, pH < 6.5) (State Bureau of Environmental Protection. GB 15618-1995), indicating that soil remediation is required. The Cd content in the soil planted is decreased by 0.05–0.08 mg/kg under different treatments (0.53 ± 0.18 for the second disposal and 0.56 ± 0.14 for the first (without any additives), and the Cd content in the soil planted without any additives is decreased most, indicating that it had the highest extraction efficiency. The Cd content in unplanted soil remained unchanged.

The extraction efficiency was 5.71%–7.01% for the aboveground part and 5.48%–6.49% for the underground part. No consideration of the effect of rainwater leaching was taken in this study; assuming that the extraction efficiency of Cd by plants to soil is fixed, the Cd content (0.61 mg/kg) of tillage soil in the test area was restored to the limit value of the second grade standard of national soil environmental quality, and calculated according to Formula (5), in the treatment without applying additives. The restoration period was the shortest, being only 8.49 years, and 10.78 years, respectively, when Treatment (2) was added. Therefore, in the low acid and Cd polluted farmland soil, there is a good potential for soil remediation without additive treatment.

### Effects of Cd on biological indicators of *P. hydridum*

Table 2 shows the correlation analysis between the biological indicators and the actual Cd concentration in soil. The number of tillers increased with the increase in Cd concentration, but R^2^ was low and the correlation was not significant. There was a significant linear negative correlation between plant height and Cd concentration, and the correlation coefficient was 0.4269, indicating that the plant height would decrease by 2.5482 cm if the Cd concentration increased by 1 mg/kg. There was a significant exponential negative correlation between stem biomass and the Cd concentration, and the correlation coefficient was 0.6539. The biomass of withered aging leaves, fresh sheaths, and aging sheaths were correlated with Cd concentration in a binomial function, with a significant correlation for fresh sheaths. There was a linear negative correlation between the Cd concentration and the fresh leaf biomass and root biomass with correlation coefficients of 0.4111 and 0.1751, respectively, showing that they were significantly correlated with the concentration of Cd in soil.

**Table 2:** Correlation between biological parameters of *P. hydridum* (y) and soil Cd concentrations (x: mg/kg).

Biological parameters	Equation type	R^2^	Equation
Tiller number	Linear	0.0732	y = 0.0458x + 3.5819
Plant height/cm	Linear	0.4269 *	y =−2.5482x + 99.199
Fresh leaf/g	Linear	0.4111 *	y = − 1.9459x + 40.401
Aging leaf/g	Binomial	0.2701	y = − 0.159x
Fresh sheath/g	Binomial	0.6001 *	y = − 0.4491x2 + 5.6798x + 6.6601
Aging sheath/g	Binomial	0.3671	y = − 0.1061x^2^ + 1.5879x + 3.0368
Stem/g	Exponential	0.6539**	y = 42.4421e − 0.071x
Shoot	Binomial	0.5798**	y = − 0.6802x^2^ + 5.0011x + 107.91
Root	Linear	0.1751	y = − 0.7502x + 40.401

Note: *  indicates a significant association (P <  0.05), and ** indicates a very significant association (P < 0.01).

The correlation analysis between the proportion of total biomass contained in each part and the actual Cd concentration in soil is shown in Table 3. The biomass proportion of green leaves, yellow leaves, and shoot parts is correlated with the Cd concentration in soil in an exponential, linear, and binomial function, respectively, but none of these reached a significant level. The biomass proportion of fresh sheath, aging sheath, and stem were correlated with the Cd concentration in soil as linearity, binomial, and logarithmic functions, respectively, with R^2^ values of 0.5191, 0.5689, and 0.4701, respectively, reaching a significant correlation.

**Table 3:** Correlation between allocation of biomass of *P. hydridum* (y, %) and soil Cd concentrations (x, mg/kg).

Biological parameters		Equation type	R^2^	Equation
Shoot biamass	Fresh leaf	Exponential	0.2589	y = 22.011x^– 0.105^
	Aging leaf	Linear	0.03301	y = 0.1719x + 12.111
	Fresh sheath	Binomial	0.5191*	y = − 0.2623x^2^ + 3.6212x + 4.5497
	Aging sheath	Binomial	0.5689*	y = − 0.0469x^2^ + 0.9487x + 2.1987
	Stem	Binomial	0.4701*	y = − 2.6109lnx + 25.301
Root biamass		Logarithmic	0.2597	y = 0.129x^2^ - 1.0691x + 26.894

Note: *  indicates a significant association (P <  0.05), and ** indicates a very significant association (P <  0.01).

### Influence of Cd content on the safe application of the method

If used as feed, fertilizer or biomass fuel substances, the materials must have different most stringent Cd maximum limit range. The most stringent Cd maximum limit value must range below 0.5mg/kg in the “Forage Hygienic Standard” (GB 13078-2001), In the Ecological Index of Arsenic, Cd, Lead, Chromium and Mercury in fertilizers (GB/T 23349-2009), the limit value of Cd and its compounds in fertilizers must be below 10 mg/kg. In the Technical Specification for Biomass Briquette Fuels and Combustion Equipment (SZDB/Z109-2014) Cd limit requirements stipulated in the biomass molding fuel soluble range below 1mg/kg. The dry/wet ratio of the *P. hydridum* in the experiment area was 0.231. According to the Cd content of the dry weight of the grass in the part of the grassland as shown in Fig 1, the Cd content of the fresh weight of the imperial bamboo grass produced by each treatment in the experiment area was 0.21-0.28mg/kg. According to the uses (feed, fertilizer, biofuel, etc.), the Cd content value obtained in this study was compared with the limit value of Cd content in relevant national standards, and it was far lower than 0.5, so it was confirmed that its application was safe.

## Discussion and conclusion

All the results showed a decrease in Cd in the contaminated soils. Under various treatment conditions, the Cd content in the soil after *P. hydridum* harvesting was 0.53–0.56 mg/kg, and the partial Cd content in the shoot was 0.21–0.28 mg/kg (fresh weight), and the enrichment coefficients were all greater than 1, and the extraction amount and efficiency of Cd were 7.17–9.43 mg/m^2^ and 5.71%–7.01%, respectively. The Cd content in the 0–20 cm topsoil layer of the experimental area (0.61 mg/kg during the research) was less than the national secondary standard of soil environmental quality (total Cd ≤ 0.30 mg/kg), and it takes 9 years at least according to the above calculation. These findings indicate that there is great potential for Cd-polluted soil treatment and remediation. Although Cd in soil is not beneficial to the growth, and the treatment with a higher Cd concentration can inhibit its growth, the *P. hydridum* is significantly correlated with the concentration of soil Cd pollutants. Moreover, the biomass increases first and then goes down with the increase in soil Cd content, indicating that a lower soil Cd content can promote the accumulation. The correlation between the biomass allocation rate of *P. hydridum* and Cd in the soil reflects the law of the material and energy allocation of Cd to its different components. After 6 months of growth, the relationship between the biomass allocation rate of fresh leaves, aging leaves, sheath, stem, and the root biomass and Cd content fitted to different models, which indicates that the biomass allocation of the plant behaved in different ways with the increase of Cd in soil. Among these, the biomass of the organs with high metabolism accounted for a larger proportion of the total biomass of the whole plant, and it was more seriously affected by Cd than that of the aged organs. The correlation coefficients (*R*^*2*^) between the Cd concentration in the fresh leaves, fresh sheath, and stem were 0.4111, 0.6001, and 0.6539, respectively, showing a significant correlation and the coefficients were higher than those of the aging organs. The stem is the most important component, the vigorous growth of which will inevitably drive the reaction of biomass accumulation rate of other components. Increased growth of the stem component increases the entire individual biomass and restrains Cd levels, which will inevitably lead to a decrease in the Cd levels in the whole plant [27].

It was reported that the Cd of *P. hydridum* in soil could be reduced by 4.6% at 2 cuttings per year, and the remediation effect on Cd contaminated soil was better than that at 1 cutting per year [28]. Although organic acids and chelates can effectively enhance the extraction efficiency of heavy metals, nitrogen fertilizer (ammonia nitrogen is the best) is more efficient in improving the enrichment efficiency of heavy metals for this plant and is more friendly to soil microorganisms [29]. These results are similar to this study. In laboratory conditions, some studies have also shown some similar effects. For example, Yi et al. (2014) [30] showed that the content of partial Cd in the soil treated with 1 mg/kg Cd on *P. hydridum* was 2.56 mg/kg, which was basically consistent with the results of this study. Li et al. [31] found that the content of partial Cd in the plant in 1 mg/kg Cd treatment water was 13.38 mg/kg, which was higher than that in this study, probably because of the higher effectiveness of Cd under hydroponic conditions and easier absorption by plants. Under the same planting conditions, the removal rate of soil Cd is 3.5 and 2.6 times that of Solanum nigrum L. and Thlaspi caerulescens L., respectively [32]. Under 0.5–100 mg/kg Cd stress, the extraction efficiency of Cd was 0.22–1.86 mg/strain [33,34]. In addition, Ma et al. (2013) [35] showed that the combined action of the plant and improvers (phosphogypsum, contaminated waste, and edible fungi) could effectively reduce heavy metals in red mud. Many experimental results reflect that the synergistic decomposition of organic matter in sludge by earthworms and microorganisms can significantly increase the available nutrients in sludge and form efficient organic fertilizers and soil amendments [36]. It is shown that earthworms promote nitrogen fixation [37], and there are more organisms in the additive growth system [38], reduce the exchange state of heavy metals and increase the content of oxidizable and residual states to improve bioavailability [39], degrade organic matter through feeding and digestion [40], and improve community structure through synergistic interaction with microorganisms [41]. Earthworm castings, especially have good soil heavy metal repair potential, rich in humic acid and microorganisms, which can effectively adsorb heavy metals, reduce the biological activity of heavy metals, and reduce the leaching migration of heavy metal ions in soil [42]. Because the plant’s own regulatory mechanism can produce soluble sugars and proline osmotic regulatory substances to relieve stress, *P. hydridum* did not show visible damage even under considerable heavy metal stress [43]. The application of earthworm manure to planting *P. hydridum* has a good effect on the ecological restoration of soil in the rare earth tailings abandoned land, which can significantly improve soil acidification, increase soil nutrient content and reduce soil heavy metal content. This is similar to the results of this study, when earthworm castings and biogas slurry were added to Treatment 2, the extraction amount and enrichment coefficient of Cd were higher. Organic fertilizers are those which contain organic matter, can provide a variety of inorganic and organic nutrients for crops, and can be fertilized to improve the soil, including human manure, manure, compost, green manure, cake fertilizer, biogas fertilizer, etc. It has a high ammonia nitrogen content, and earthworm castings in particular can make the soil rich in humic acid, which can both improve the acid-base balance of the soil and play a role in thinning the soil as an accelerant in plant growth, conducive to crop growth. Compared with fertilizer, its most important characteristics are: rich in balanced nutrients, long-term fertilizer effect, can be very good land cultivation, the growth of crops, but also to provide a great help. Soil amendments are only an accelerant in plant growth, which can improve the acid-base balance of the soil and play a role in thinning the soil, which is conducive to crop growth. The main improvers in this study are lime and sodium hydroxide. From a safety perceptive, results of this study shows that, the Cd content of *P. hydridum* produced by various treatments in polluted areas can be safely used in livestock feed, fertilizer, or biomass fuel, and the Cd content is within the limiting range of relevant standards. Mei [44] used *P. hydridum* and amendments to jointly control the lead-zinc mine tailings contaminated agricultural soils in the Dahuanjiang River basin of Guangxi in China, and found that the soil Cd content could be reduced to the secondary soil environmental quality standard in only 1.2 years, and it was safe and feasible to feed it to beef cattle or other animals.

In this field plot experiment, *P. hydridum* was conducted to study its field treatment and restoration effect on Cd-polluted farmland under the condition of different soil additives, as well as the influence of Cd on the biological indicators of the grass, and the safety of its subsequent application was also analyzed. It will provide reference for the treatment, restoration and safe application of Cd- pollution soil. The high biomass and heavy metal tolerance determine the potential of remediation of heavy metal contaminated soil. Suitable agronomic measures can effectively promote the growth of *P. hydridum* or increase the bioavailability of heavy metals in soil, which is an effective measure to improve the phytoremediation efficiency of heavy metal contaminated soil [45]. In this study, the grass of the experimental land grew well, which may be due to the fact that earthworm castings reduced the activity of heavy metals in the soil and provided sufficient fertility for their growth. *P. hydridum* has strong resistance to stress, wide adaptability, and low requirements in terms of its environment. It can survive and grow well in areas with harsh conditions such as heavy metal pollution, salinization, and soil erosion. It is important to make full practical use of the excellent characteristics of the drought resistance, toleration of barren conditions, salt resistance, acid resistance, and lodging resistance. These characteristics can not only improve the regional ecological environment of the plant, but also give full play to the productive potential of degraded soil, so as to realize the production of biomass raw materials. It can be safely applied to forage grass, returning fertilizer, papermaking raw materials, energy plants, etc., which expands the comprehensive application prospect of polluted farmland. In addition, many studies have shown that it has a good application prospect in environmental management such as soil and water conservation and vegetation restoration [46]. The complex ecological economic model combining agriculture, forestry and forage is an effective way of sustainable development, especially in the area with unfavorable ecological environment or in the project of returning farmland to forest and grassland. Systematic research and exploration of its application in ecological environment governance and restoration, and the development of its supporting industrial chain are of great significance to the implementation of returning farmland to forest and grassland and the construction of ecological barriers. It is also conducive to the development of a new rural model combining planting and breeding, integrating the ecological economy, and improving the livelihoods of farmers in poor mountainous areas. Although there are many researches on its application in drought resistance, salinization resistance, sewage treatment and other ecological environment management, there are few reports on its corresponding physiological plant characteristics. This will greatly restrict its ecological application value. Therefore, the physiological mechanism of using it to control ecological environment and survival under adversity needs further study, as does the ecological consequences of controlling invasive species. The relationship between soil Cd availability and Cd absorption and enrichment has not been studied in this paper, and future studies should be further studied to improve the restoration effect by applying more appropriate additives and other auxiliary measures.

In summary, *P. hydridum* is a hyperaccumulating plant that can tolerate many heavy metal stresses. Appropriate agronomic measures can effectively improve the heavy metal enrichment efficiency and realize its ability to remediate heavy metal contaminated soil. Moreover, it can be safely applied as forage grass, field return fertilizer, papermaking raw materials, or energy biomass production, expanding the potential application prospects of polluted farmland.

## Supporting information

S1 File Supplementary data. (DOCX)

## References

[1] Lane TW, Morel FM. A biological function for cadmium in marine diatoms. Proc Natl Acad Sci U S A. 2000;97(9):4627–31. doi: 10.1073/pnas.090091397 10781068 PMC18283

[2] DalCorso G, Farinati S, Maistri S, Furini A. How plants cope with cadmium: staking all on metabolism and gene expression. J Integr Plant Biol. 2008;50(10):1268–80. doi: 10.1111/j.1744-7909.2008.00737.x 19017114

[3] Alcantara E, Romera FJ, Canete M, De la Guardia MD. Effects of heavy metals on both induction and function of root Fe (III) reductase in Fe-deficient cucumber (Cucumis sativus L.). J Exp Bot. 1994;45:1893–8.

[4] Pahlson AB. Toxicity of heavy metals (Zn, Cu, Cd, Pb) to vascular plants. Water Air Soil Pollut. 1989;47:287–319.

[5] Schützendübel A, Schwanz P, Teichmann T, Gross K, Langenfeld-Heyser R, Godbold DL, et al. Cadmium-induced changes in antioxidative systems, hydrogen peroxide content, and differentiation in Scots pine roots. Plant Physiol. 2001;127(3):887–98. doi: 10.1104/pp.010318 11706171 PMC129260

[6] Romero-Puertas MC, Palma JM, Gomez M, Del Rio LA, Sandalio LM. Cd causes oxidative modification of proteins in pea plants. Plant Cell Environ. 2002;25 677–86.

[7] Benavides MP, Gallego SM, Tomaro ML. Cd toxicity in plants. Braz J Plant Physiol. 2005;77:21–34.

[8] Kovalchuk I, Titov V, Hohn B, Kovalchuk O. Transcriptome profiling reveals similarities and differences in plant responses to cadmium and lead. Mutat Res. 2005;570(2):149–61. doi: 10.1016/j.mrfmmm.2004.10.004 15708574

[9] Clemens S. Evolution and function of phytochelatin synthases. J Plant Physiol. 2006;163(3):319–32. doi: 10.1016/j.jplph.2005.11.010 16384624

[10] Alfvén T, Elinder CG, Carlsson MD, Grubb A, Hellström L, Persson B, et al. Low-level cadmium exposure and osteoporosis. J Bone Miner Res. 2000;15(8):1579–86. doi: 10.1359/jbmr.2000.15.8.1579 10934657

[11] Hellström L, Elinder CG, Dahlberg B, Lundberg M, Järup L, Persson B, et al. Cadmium exposure and end-stage renal disease. Am J Kidney Dis. 2001;38(5):1001–8. doi: 10.1053/ajkd.2001.28589 11684553

[12] Wang H, Zhu G, SHi Y, Weng S, Jin T, Kong Q, et al. Influence of environmental Cd exposure on forearm bone density. Influence of environmental Cd exposure on forearm bone density. J Bone Miner Res. 2003;553–60. 12619941 10.1359/jbmr.2003.18.3.553

[13] Nawrot T, Plusquin M, Hogervorst J, Roels HA, Celis H, Thijs L, et al. Environmental exposure to cadmium and risk of cancer: a prospective population-based study. Lancet Oncol. 2006;7(2):119–26. doi: 10.1016/S1470-2045(06)70545-9 16455475

[14] Nordberg GF, Nogawa K, Nordberg M. Cd. In Handbook on the Toxicology of Metals, 2015; pp: 667–716.

[15] Yu K, Meng Q, Zhou J. Effects of Cd on growth, chlorophyll content and cell ultrastructure of maize seedlings. J North China Agron. 2010;25(3):118–23.

[16] Vallee BL, Ulmer DD. Biochemical effects of mercury, cadmium, and lead. Annu Rev Biochem. 1972;41(10):91–128. doi: 10.1146/annurev.bi.41.070172.000515 4570963

[17] Adesodun JK, Atayese MO, Agbaje TA, Osadiaye BA, Mafe OF, Soretire AA. Phytoremediation potentials of sunflowers (Tithonia diversifolia and Helianthus annuus) for metals in soils contaminated with zinc and lead nitrates. Water Air Soil Pollut. 2009;207:195–201.

[18] Wang X, Jia YF. Study on the absorption and remediation of heavy metals by larch in soil. Ecological Environment. 2007;16(2).

[19] Guo Y. The heavy metal cadmium and vanadium accumulation characteristics and migration behavior research in soil and alfalfa[D]. Xinjiang University, 2015.

[20] Wang X, Jia YF. Study on the absorption and remediation of heavy metals by larch in soil. Ecol Environ. 2007;16(2): 432–6.

[21] Li Q, Xiao Y, He J. Effects of Cd polluted soil on the growth and accumulation of P. hydridum. Jiangsu Agric Sci. 2012;40(11):354–6.

[22] Li H, Liu Y, Lu HW. On the effects influencing the growth of the two rice varieties due to Cd contaminated soil passivation in Hunan. J Saf Environ. 2016;16(6):298–302.

[23] Zhong Y, Lei X, Li J. Effect of earthworm droppings planting P. hydridum on soil improvement of rare earth tailings. Jiangxi Sci. 2016;34(2):37–40.

[24] Zi C, Wang YH, Jing S, et al. Analysis of water environment risk on biogas slurry disposal in paddy field. Trans Chin Soc Agric Eng. 2016;32(5):213–20.

[25] Ma CJ, Jia CS, Li HS. Influences of P. hydridum plantation on the edaphon in different site conditions[C]. 2014 2^nd^ International Conferences on Social Science and Health (ICSSH 2014). Information Engineering Research Institute, 2014: 222–26.

[26] Wang XN, Yi ZC, Zhang YF. Response of P. hydridum to heavy metal contaminated chicken manure and sludge and its remediation effect. J Agric Resour Environ. 2015;32(5):477–84.

[27] Ma CJ, Liu FG. Research progress on the application of imperial P. hydridum in ecological environment management. Soil Water Conserv China. 2012;33(1): 41–4.

[28] Ishii Y, Hamano K, Kang DJ. Cd phytoremediation potential of napiergrass cultivated in Kyushu, Japan. Appl Environ Soil Sci. 2015;15(1):1–6.

[29] ZHang X, Gao B, et al. Effect of Cd on growth, photosynthesis, mineral nutrition and metal accumulation of an energy crop, king grass (Pennisetum americanum × P. purpureum). Biomass Bioenergy. 2014;67:179–87.

[30] Zi-cheng Y, Jun-bo H, Hua CH. Effects of Cd polluted soil on the modular growth and physiological characteristics of P. hydridum. J Agro-Environ Sci. 2014;33(2):276–82.

[31] Li Q, Xiao Y, He J. Effects of Cd polluted soil on the growth and accumulation of P. hydridum. Jiangsu Agric Sci. 2012;40(11):54–356.

[32] Zhang X, Xia H, Li Z, Zhuang P, Gao B. Potential of four forage grasses in remediation of Cd and Zn contaminated soils. Bioresour Technol. 2010;101(6):2063–6. doi: 10.1016/j.biortech.2009.11.065 20005700

[33] Zhang X, Zhang X, Gao B, et al. Effect of Cd on growth, photosynthesis, mineral nutrition and metal accumulation of an energy crop, king grass (Pennisetum americanum × P. purpureum). Biomass and Bioenergy. 2014;67:179–87.

[34] Hu L, Wang R, Liu X, Xu B, Xie T, Li Y, et al. Cadmium phytoextraction potential of king grass (Pennisetum sinese Roxb.) and responses of rhizosphere bacterial communities to a cadmium pollution gradient. Environ Sci Pollut Res Int. 2018;25(22):21671–81. doi: 10.1007/s11356-018-2311-9 29785604

[35] Ma C, J M, Li HS. Study of red mud improvement with the mixing method and the impact of Pennisetum hybridum plantation on red mud amendment. Adv Mater Res. 2013;807 392–401.

[36] Huang Y, Ou L. Application of earthworm compost to sludge. J Agric Technol. 2019;39(23):133–4.

[37] Shao YH, Zhang WX, Liu SJ. Soil animal diversity and its ecological function. Acta Ecol Sin. 2015;35(20):6614–25.

[38] Na L, Li Y, He J. Effects of earthworm on nitrogen uptake and nitrogen fixation by microorganisms after nitrogen application. J Agric Environ Sci. 2019;39(2):34–350.

[39] Lu F, Wang X, Chu Z. Effect of earthworm on bioavailability of heavy metals in reclaimed soil with different thicknesses. Chin J Ecol. 2019;41(1):124–31.

[40] Yang J, Sang CL, Huang K. Effect of earthworm inoculation on removal of organic matter from residual sludge in vertical flow wetland. Environ Pollut Prev. 2019;44(11):1422–8.

[41] Su Q, Li LF, Zhu CX. Effects of earthworm/cerium-manganese modified biochar on bacterial diversity and community structure in red soil polluted by As. J Environ Sci. 2012;43(3):1630–40.

[42] Chen XJ. Study on the improvement effect of earthworm manure on red soil after earthworm digestion of cow dung. Yangzhou: Yangzhou University, 2016.

[43] Gallego SM, Pena LB, Barcia RA. Unravelling Cd toxicity and tolerance in plants: Insight into regulatory mechanisms. Environ Exp Bot. 2012;83:33–46.

[44] Mei J. Remediation and utilization of lead-zinc mine tailings contaminated agritultural soils. Taiyuan: Shanxi University, 2013.

[45] Cui H, Fan Y, Yang J, Xu L, Zhou J, Zhu Z. In situ phytoextraction of copper and cadmium and its biological impacts in acidic soil. Chemosphere. 2016;161:233–41. doi: 10.1016/j.chemosphere.2016.07.022 27434253

[46] He L, Zhu Q, Wang Y, He M, Tan F. Advances in research on the comprehensive utilization of a perennial grass Pennisetum hydridum. Chin J Appl Environ Biol. 2020;26(3):705–12.

