# Supplementary material for: Analysis on the potential of Pennisetum hydridum for phytoremediation of Cd-polluted soil fertilized by worm castings
Source: PLoS One. 2025 Mar 31;20(3):e0318528. doi: 10.1371/journal.pone.0318528 (PMC11957277; doi:10.1371/journal.pone.0318528)
Supplement: S1 File — (DOCX) [file pone.0318528.s001.docx]

**2021.10.18-2022.11.18 Experimental design and sampling data organization**

**I. Experimental design and sample collection**

1. Overview of experimental area site selection

The test area belongs to the Pennisetum hydridumis base of Jingnong Husbandry Ecological Agriculture Demonstration Park, located in Zhutian Township, Suichuan County, Ji'an City, Jiangxi Province, geographic coordinates of latitude 25°28′32″~26°42′55″, longitude 113°56′51″ ~ 114°45′45″, is a humid monsoon climate of the Central Subtropical, a mild climate, abundant rainfall, sunshine, the four seasons, winter and summer, a long time, spring and autumn is short, a long period of frost-free period,. The climate varies greatly within the territory. The average annual temperature ranges from 15.1 to 18.1℃, the average annual precipitation is 1,421.2mm, and the average annual frost-free period is 284 days. The land is fertile, with rice soil type dominating the paddy fields and red soil type in the mountains.

Detailed properties of soil conditions in the test area are shown in Table 3: pH was 6.26±0.22, acidic; soil whole Cd was (0.61±0.21) mg/kg, exceeding the second level limit value of Soil Environmental Quality Standard (GB 15618-1995) (soil whole Cd≤0.3 mg/kg, pH<6.5), which was a mild Cd pollution.

2. Experimental design

On May 18, 2022, within the same planting site, the Pennisetum hydridum with about 30d of seedling, relatively uniform growth, plant height of 35-40cm, number of true leaves of 4-5, no tillers, with little difference in specifications, and retaining stem segments with two complete stem nodes was selected as the test material.

Using field positioning test, set up two test plots, respectively, for the planting of Pennisetum hydridum, each plot area of about 667m2, planting area of 10hm2, just to facilitate the workload of a harvester. Two groups of soil additive application treatments were set up in each plot, namely (1) original soil (control), (2) application of vermicompost. Vermicompost was applied as a base fertilizer (20 kg/m2) and watered with biogas. Two replications were set up for each group of treatments, and the orientation of the treatments in the plots was randomly distributed. The soil in the experimental area was applied with earthworm manure as a base fertilizer after land leveling, tilling, and zoning in November before the onset of winter in the previous year, and the seedlings were transplanted on May 18, 2022, with about 30 d of royal bamboo grass. In order to facilitate mechanized harvesting, the planting density (row spacing × plant spacing) was 80 cm × 60 cm, and 130 seedlings were planted per mu, with an average of 4 plants in 1 m2. The experimental period was 6 months (May 2022 to November 2022), no mowing was carried out during the experimental period, no fertilization was carried out during the whole life span of the plants, the irrigation water was in accordance with the Water Quality Standard for Agricultural Irrigation (GB 5084-2005), and the weeds in the field were removed by manual weeding.

Lime: from local lime plant, pH 13, full Cd content is 0.21mg/kg.

Sodium hydroxide: from Xinjiang Zhongtai Chemical Co., Ltd. with a full Cd content of 1.19 mg.kg.

Earthworm manure: provided by Ganzhou Ruijin JASPER Earth-worm Vermiculture Co. Ltd. as cow dung cultured earthworm manure, pH 7.69, full Cd content of 1.09 mg/kg.

Methane liquid: methane liquid after producing methane gas from cattle farm effluent.

3. Sample collection

After 6 months (November 2022), because no mowing was done during this period, the plants were basically over 2 meters in height, and the diameter of each plant averaged about 60 cm with more than 30 tillers. In each area using the five-point method of sampling, collection of each sample point within 1m2 4 plants, the plant body aboveground and underground part of the separation, and then dug out the whole pocket of the Pennisetum hydridum, began to sample and analyze, and the determination of the indicators, and the rest of the harvester crushed one harvest. Among them, the aboveground part into green leaves, withered yellow leaves (more than 2/3 withered yellow), green leaf sheaths, withered leaf sheaths, stems and other five parts, composed of mixed samples; underground part of the tap water to clean the surface of the adherent soil, and then deionized water drenched 3 times, drying. Selected part of the sample, with drying method (105 ℃ kill 30min, 65 ℃ drying to constant weight) converted to the dry matter mass of each part. After tilling the soil and plant harvesting, soil samples were collected from each area according to the five-point method, and soil samples consisting of mixed samples were taken from 0~20 cm of the tillage layer, numbered at the sampling site, air-dried indoors, crushed after removing stones, plant roots and apomictic material in the soil, and sieved through 30 mesh (0.6 mm) for determining the Cd content of the soil. Soil samples were digested with HNO_3_-H_2_O_2_, plant samples were digested with HNO_3_-HClO_4_, and the method of GB/T 5009.15-2003 was used for the determination of soil heavy metal Cd.

**II. Sampling and collation data prior to grading on October 18, 2021**

Table 1 Vermicompost, biogas pH and main chemical composition (on dry weight basis)

| fertilizer | PH | Moisture content% | Organic matter content% | Total N content% | Total P content% | Total K content% |
| --- | --- | --- | --- | --- | --- | --- |
| Earthworm manure (cattle manure farming) | 7.41 | 49.88 | 40.78 | 1.79 | 3.23 | 2.24 |
| cattle digestate | 7.23 |  | 0.81 | 0.67 | 0.09 | 0.08 |

Table 2 Heavy metal composition and content of vermicompost and digestate

| fertilizer | Se(mg/kg) | Cu(mg/kg) | Pb(mg/kg) | Cd(mg/kg) |
| --- | --- | --- | --- | --- |
| Earthworm manure (cattle manure farming) | 0.45 | 87.96 | 0.12 | 1.11 |
| cattle digestate | 0.01 | 0.13 | 0.02 | 0.01 |

**III. Pre-transplant sampling data from May 10, 2022**

Table 3 Changes in soil chemical properties before and after planting

| deal with | PH | organic matter content g/kg | Total N content g/kg | Total P content g/kg | Total K content g/kg | Cd(mg/kg) |
| --- | --- | --- | --- | --- | --- | --- |
| pre-plant | 6.26±0.2 | 2.41±0.2 | 2.38±0.2 | 74.21±0.2 | 115.42±0.2 | 0.61±0.02b |
| control subjects | 5.35±0.1 | 2.19±0.2 | 2.02±0.1 | 70.12±0.1 | 102.01±0.1 | 0.53±0.02a |
| Group II | 5.49±0.5 | 2.22±0.6 | 2.17±0.3 | 68.59±0.6 | 106.09±0.3 | 0.56±0.02a |

**IV. harvest sampling data for November 18, 2023**

Table 4 Survival rate and production of C. albicans

| deal with | Survival rate % | Total fresh grass production（t/hm^2^） | Dry weight of above-ground portion of yield(kg/hm^2^) | Underground part yield dry weight（kg/hm^2^） |
| --- | --- | --- | --- | --- |
| control subjects | 67.29±0.27 | 85.79±1.12 | 5.71±2.52 | 2.35±7.01 |
| Group II | 91.07±0.14 | 117.56±1.48 | 7.91±3.18 | 3.95±4.31 |

Table 5 Main nutrient composition and content of Pennisetum hydridum

| deal with | Crude protein content % | Crude fiber content % | Crude ash content % | Crude fat content % | Ca content % | P content % |
| --- | --- | --- | --- | --- | --- | --- |
| control subjects | 7.52±0.57 | 33.33±1.04 | 10.82±0.35 | 3.12±0.35 | 0.21±0.01 | 0.11±0.01 |
| Group II | 8.23±0.51 | 32.98±1.52 | 7.34±0.24 | 1.51±0.02 | 0.14±0.03 | 0.12±0.01 |

Table 6 Content of Cd in Pennisetum hydridum

| fertilizer | ground segment (mg/kg) | underground section (mg/kg) |
| --- | --- | --- |
| control subjects | 1.65±0.31 | 1.73±0.11 |
| Group II | 0.93±0.13 | 1.02±0.44 |
